# Supplementary material for: Psychosocial working conditions as determinants of slips and lapses, and poor social interactions with patients among medical assistants in Germany: A cohort study
Source: PLoS One. 2024 Apr 16;19(4):e0296977. doi: 10.1371/journal.pone.0296977 (PMC11020507; doi:10.1371/journal.pone.0296977)
Supplement: S1 Fig — (PDF) [file pone.0296977.s001.pdf]

Figure S1. Scatterplots of psychosocial working conditions (exposures) and slips and lapses as well as interaction with patients (outcomes).

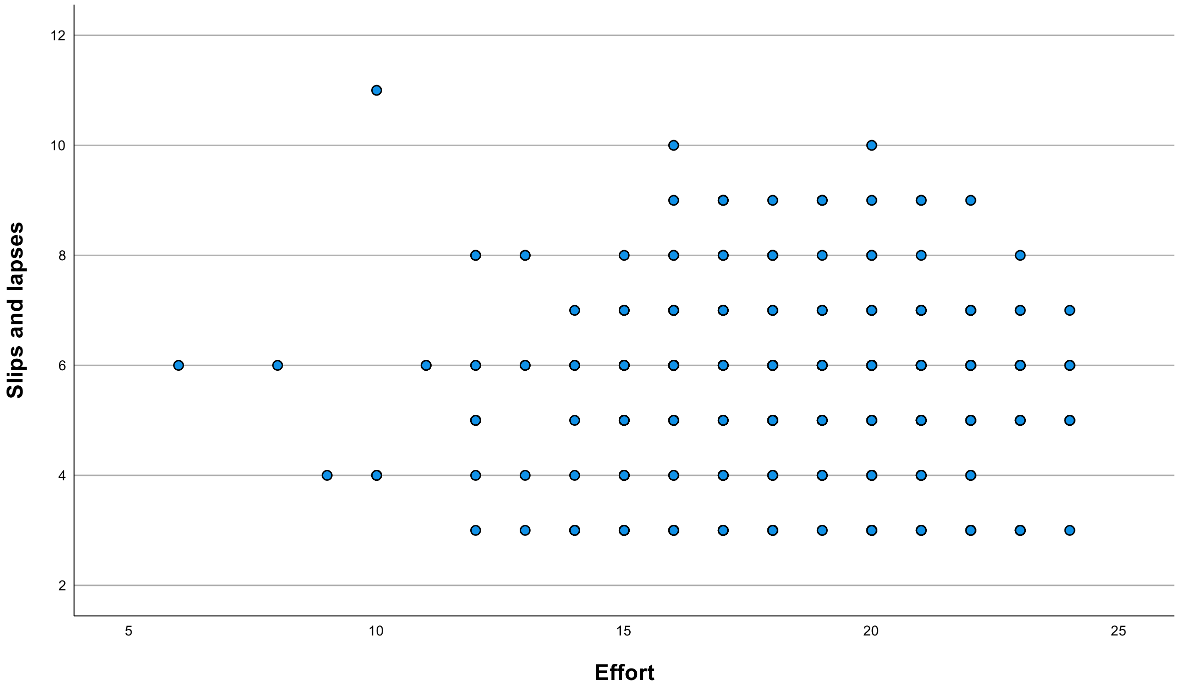

Figure S1.1. Scatterplot between effort and slips and lapses.

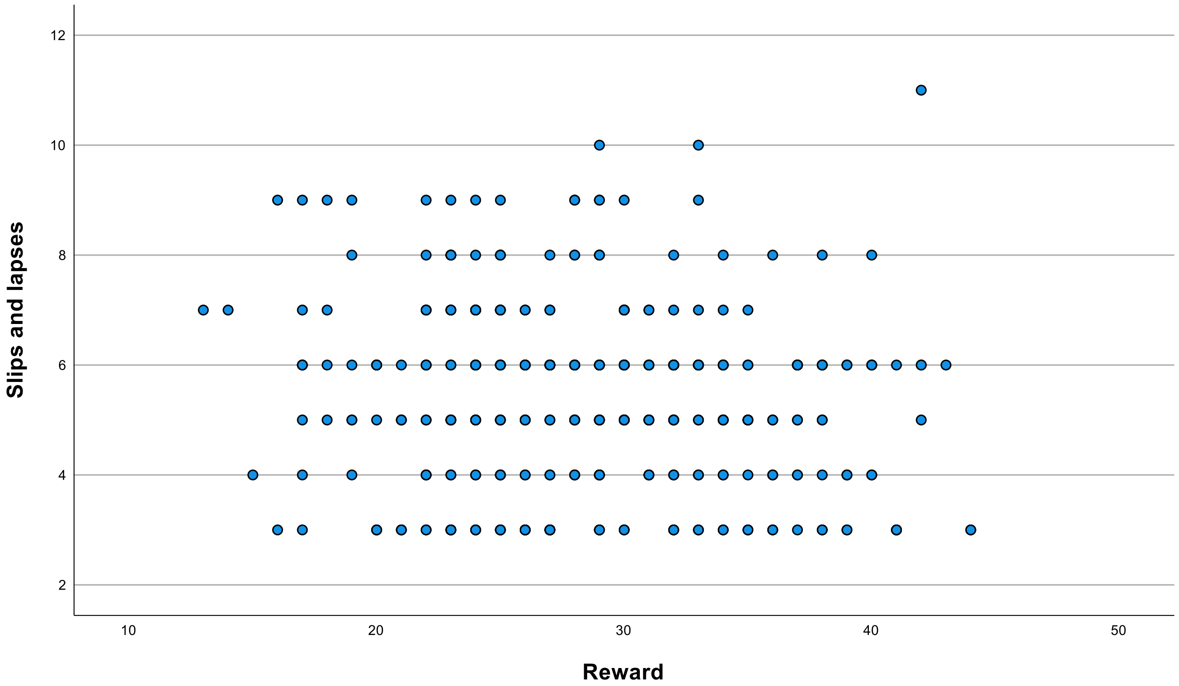

Figure S1.2. Scatterplot between reward and slips and lapses.

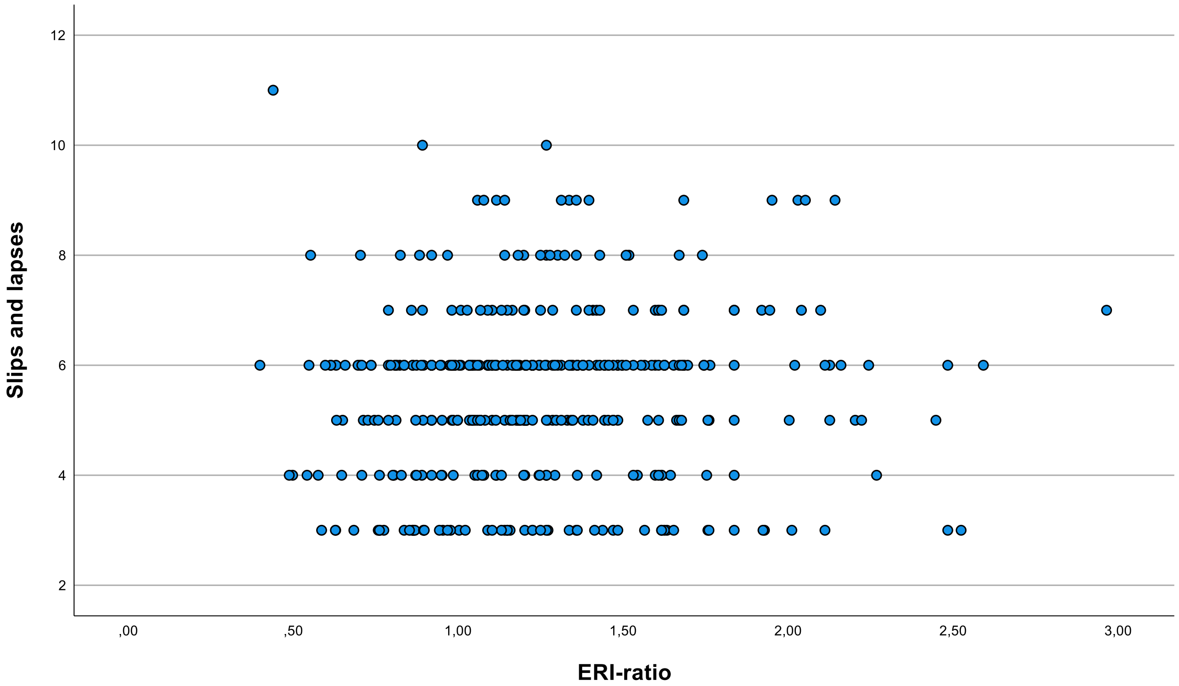

Figure S1.3. Scatterplot between effort-reward imbalance questionnaire (ERI)-ratio and slips and lapses. ERI=(Effort*11)/(Reward*6).

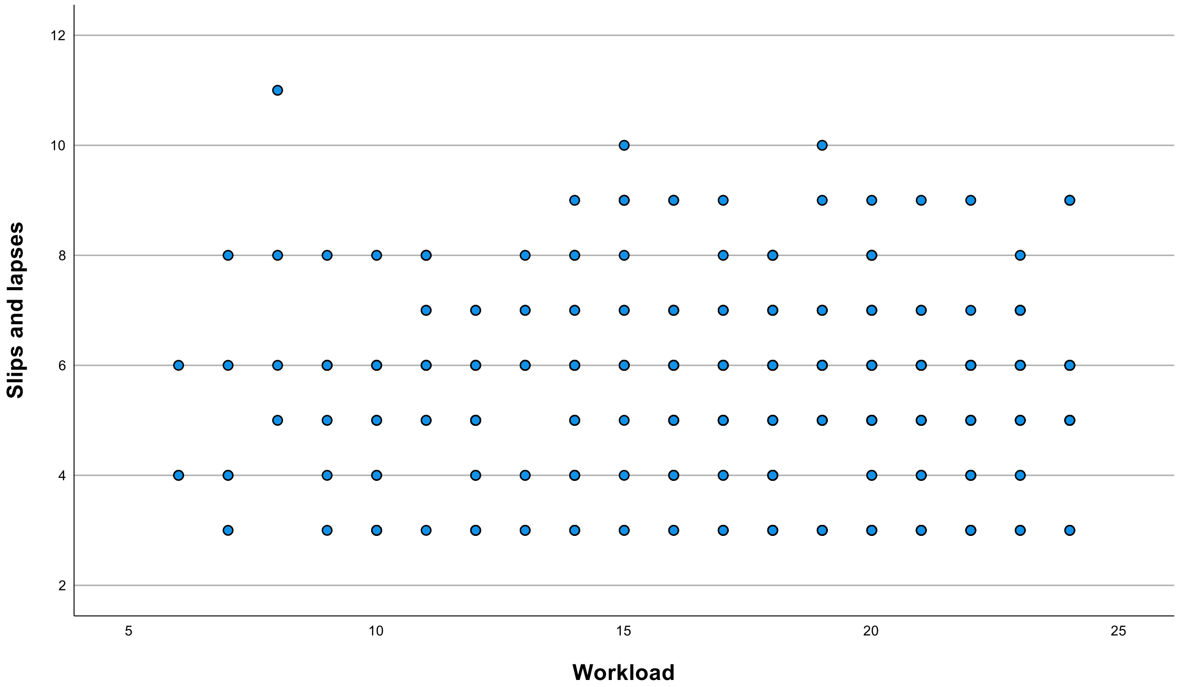

Figure S1.4. Scatterplot between workload and slips and lapses.

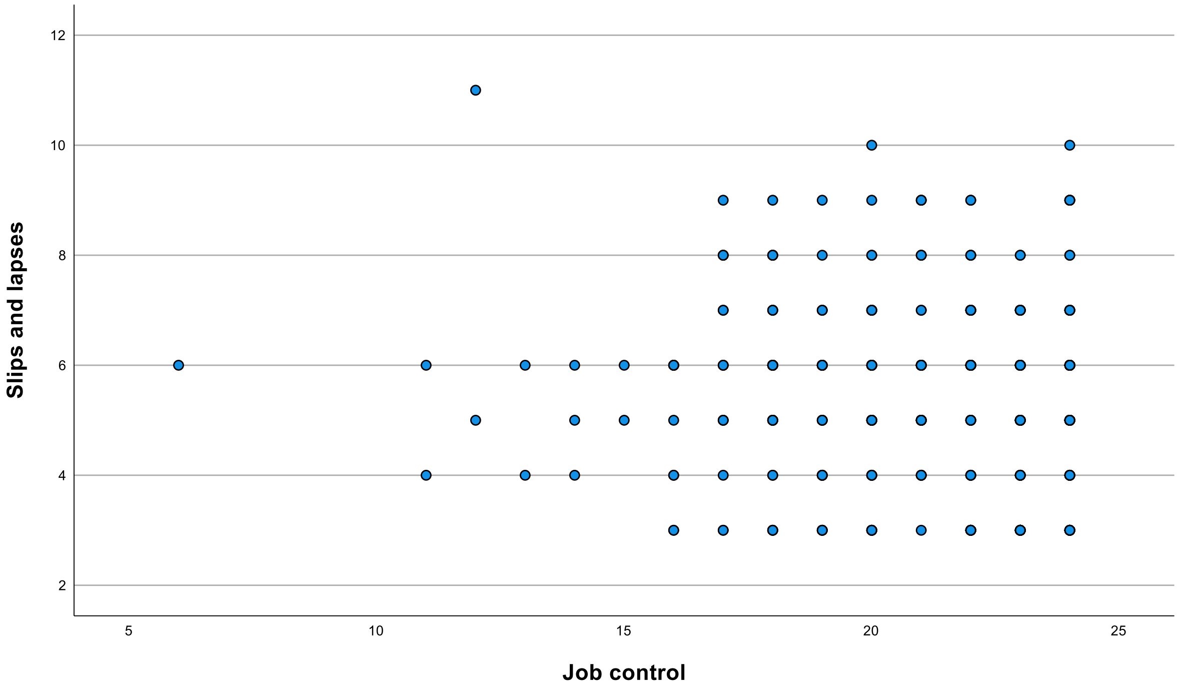

Figure S1.5. Scatterplot between job control and slips and lapses.

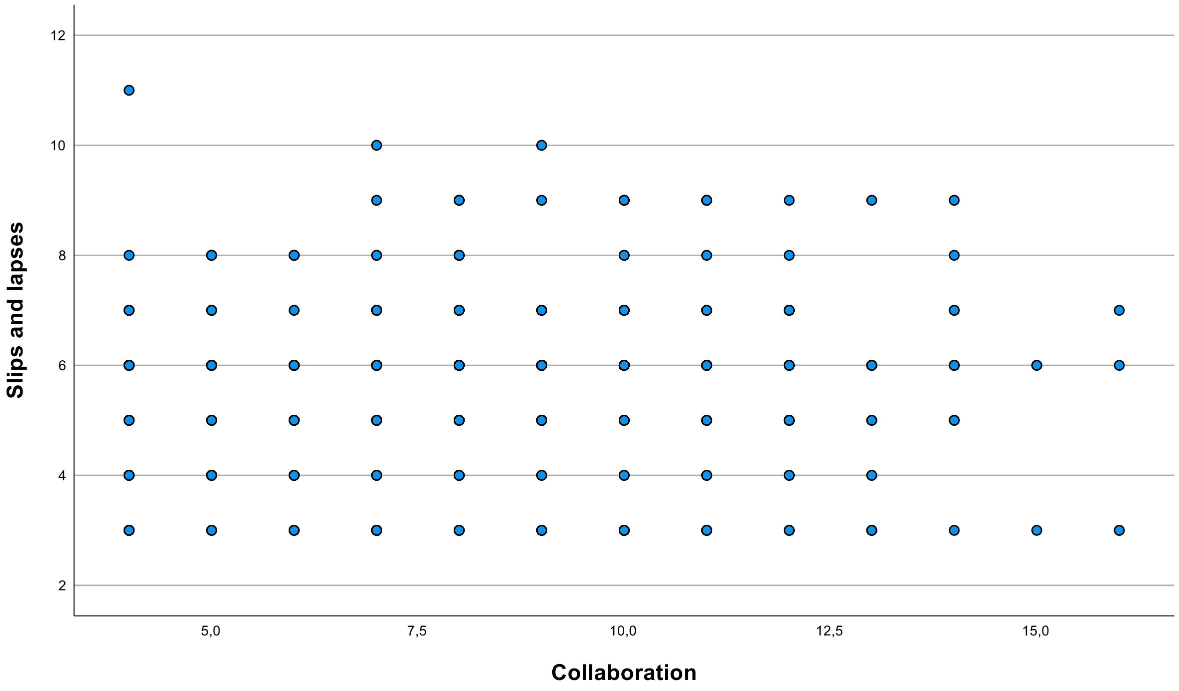

Figure S1.6. Scatterplot between collaboration and slips and lapses.

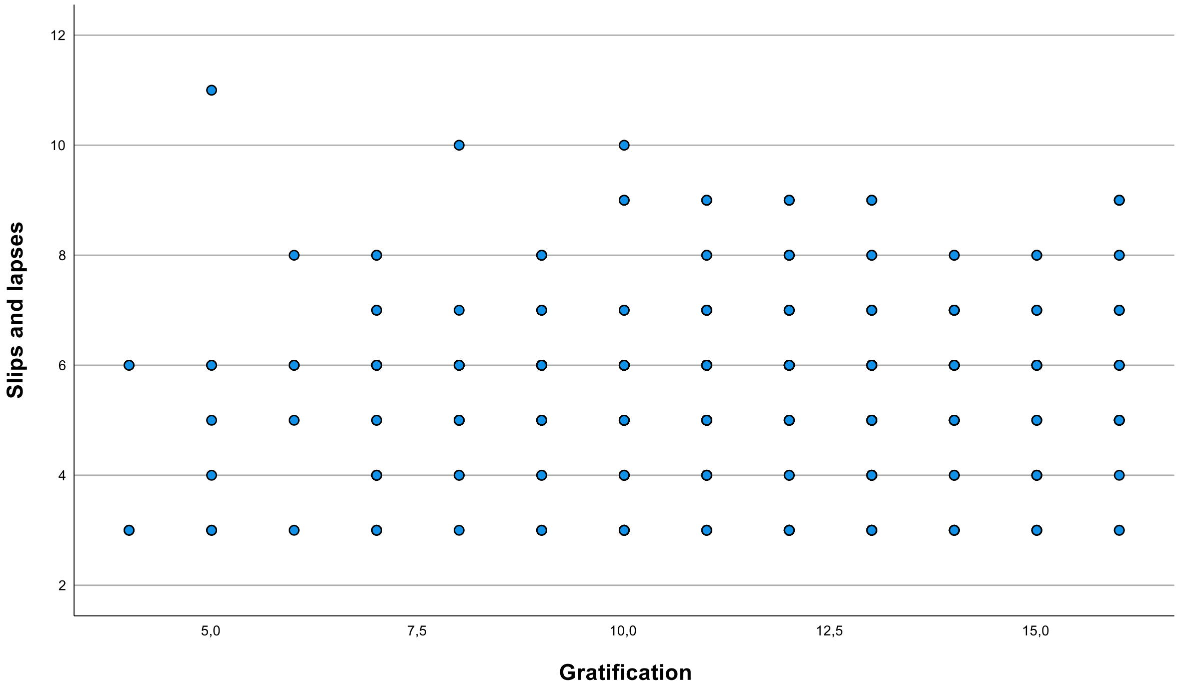

Figure S1.7. Scatterplot between gratification and slips and lapses.

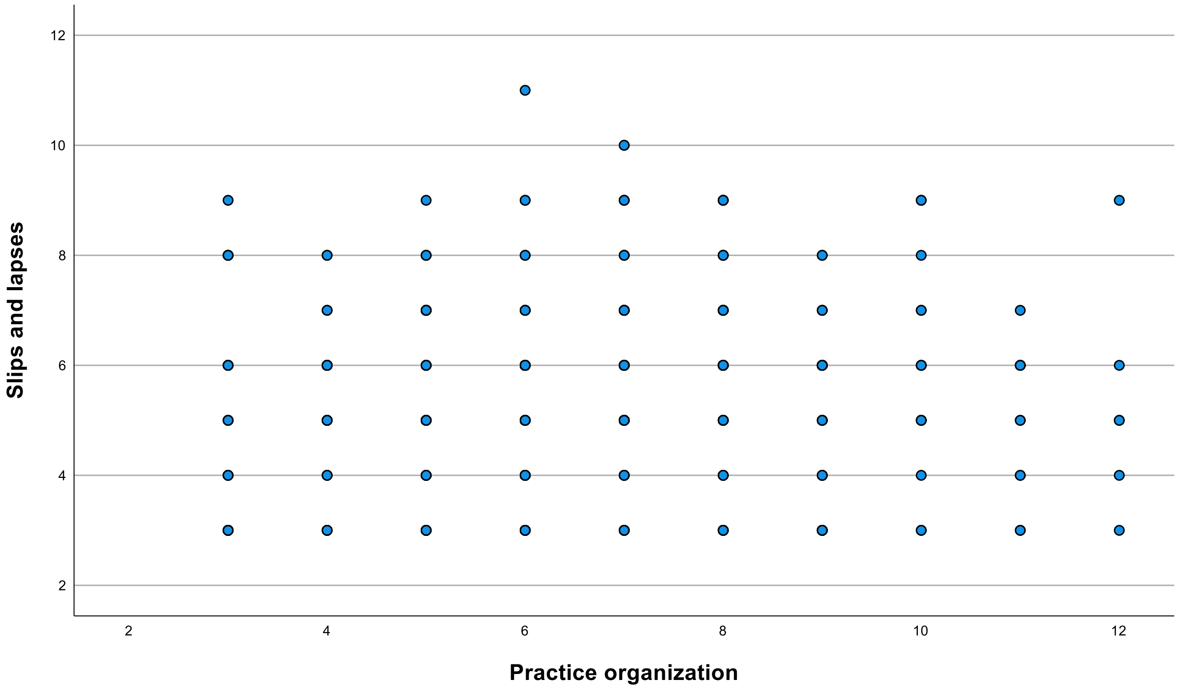

Figure S1.8. Scatterplot between practice organization and slips and lapses.

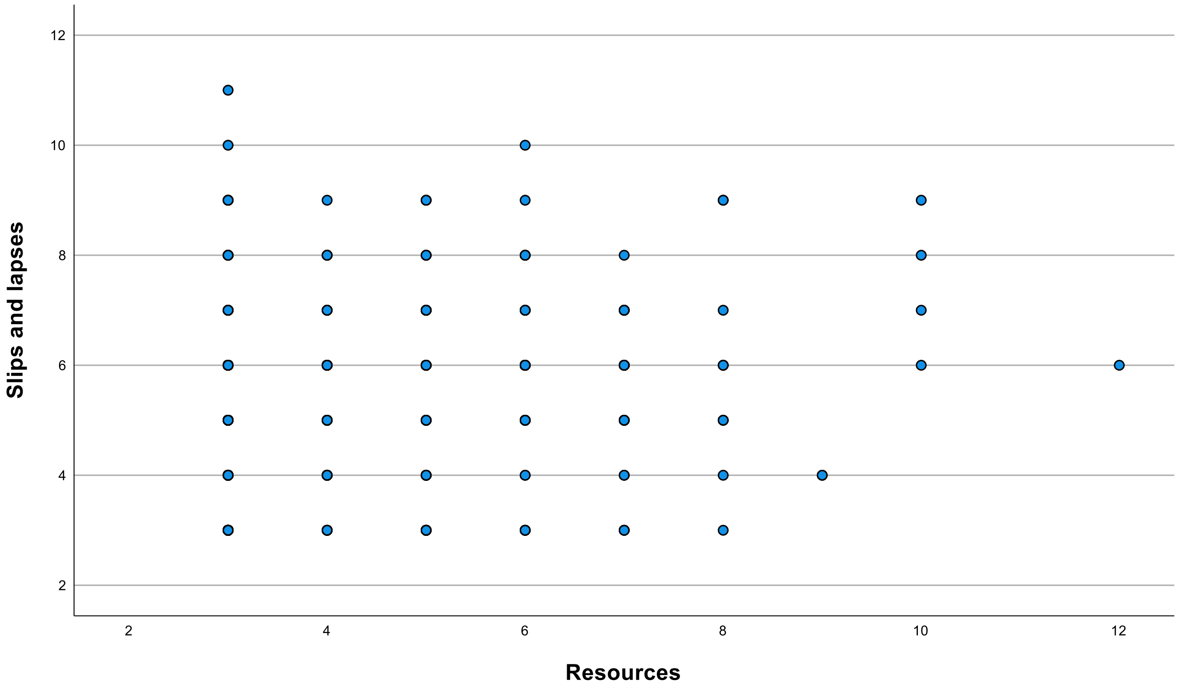

Figure S1.9. Scatterplot between resources and slips and lapses.

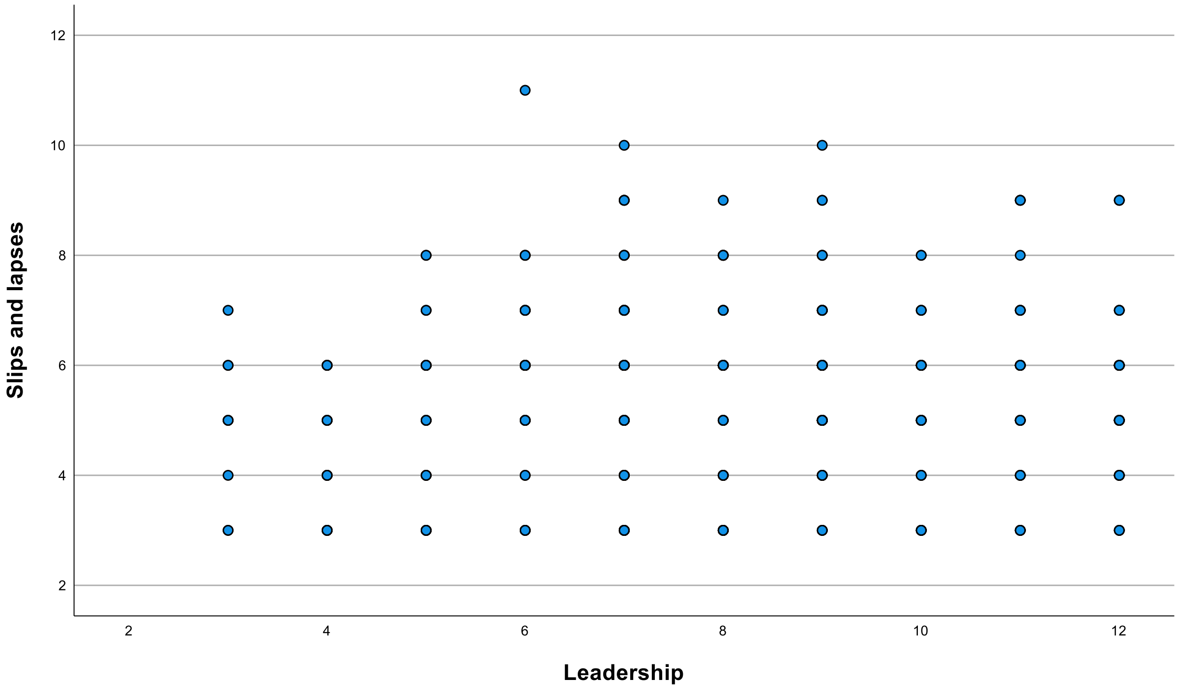

Figure S1.10. Scatterplot between leadership and slips and lapses.

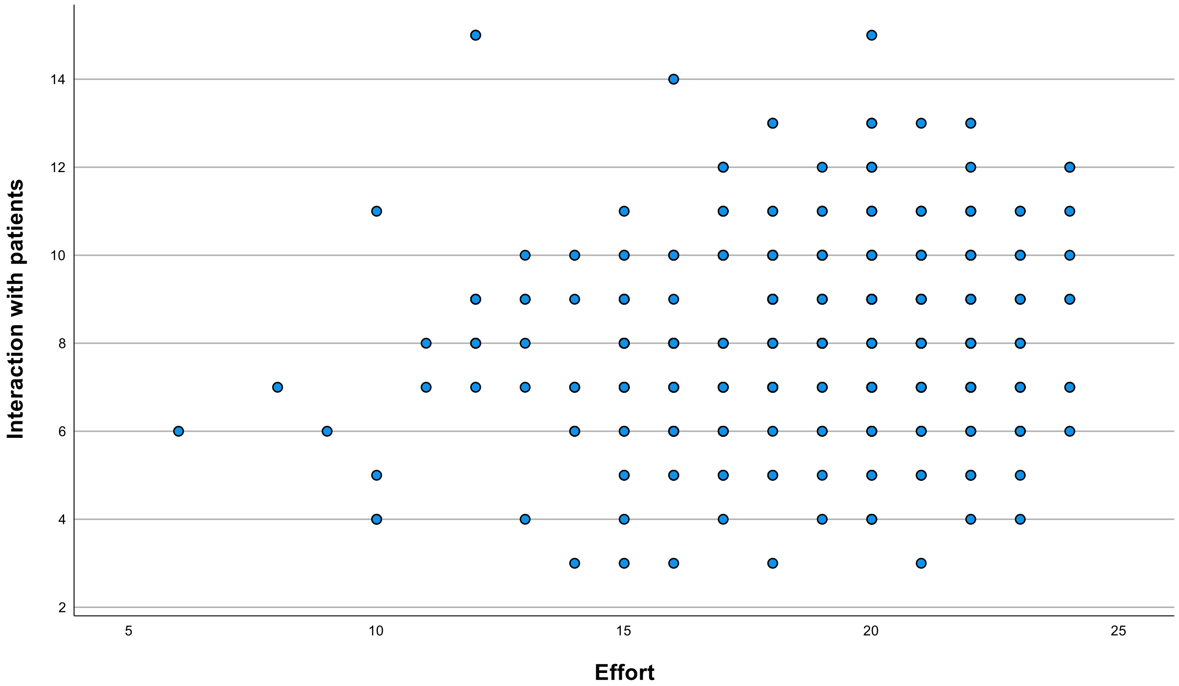

Figure S1.11. Scatterplot between effort and poor interaction with patients.

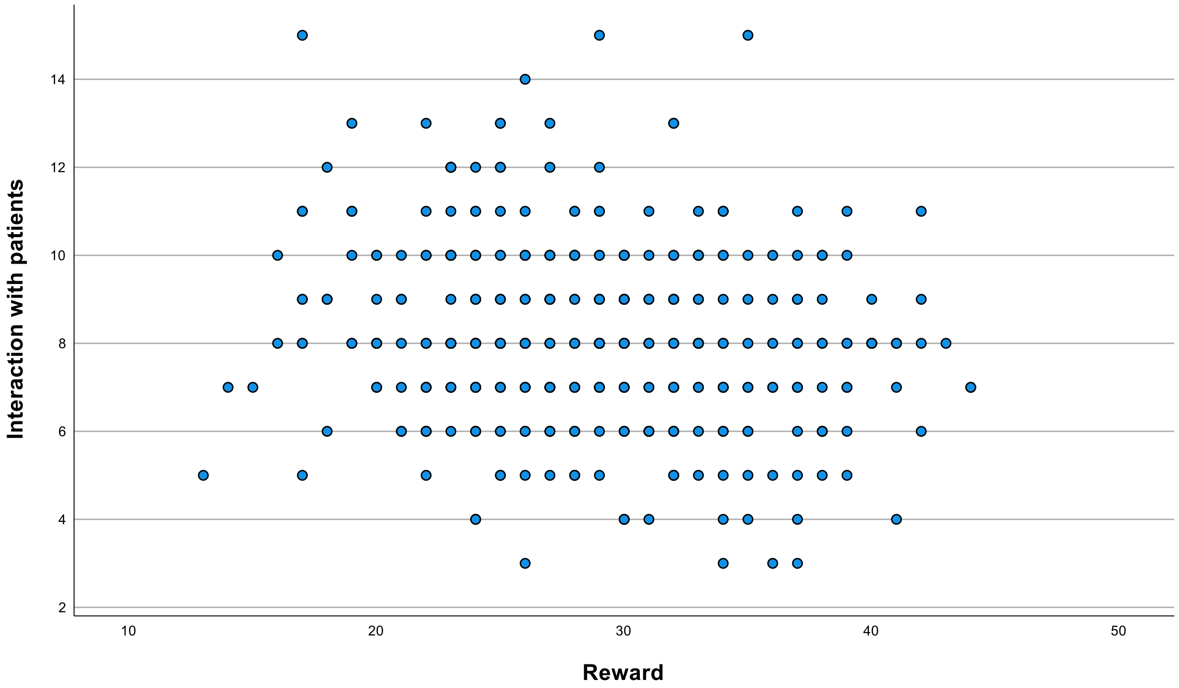

Figure S1.12. Scatterplot between reward and poor interaction with patients.

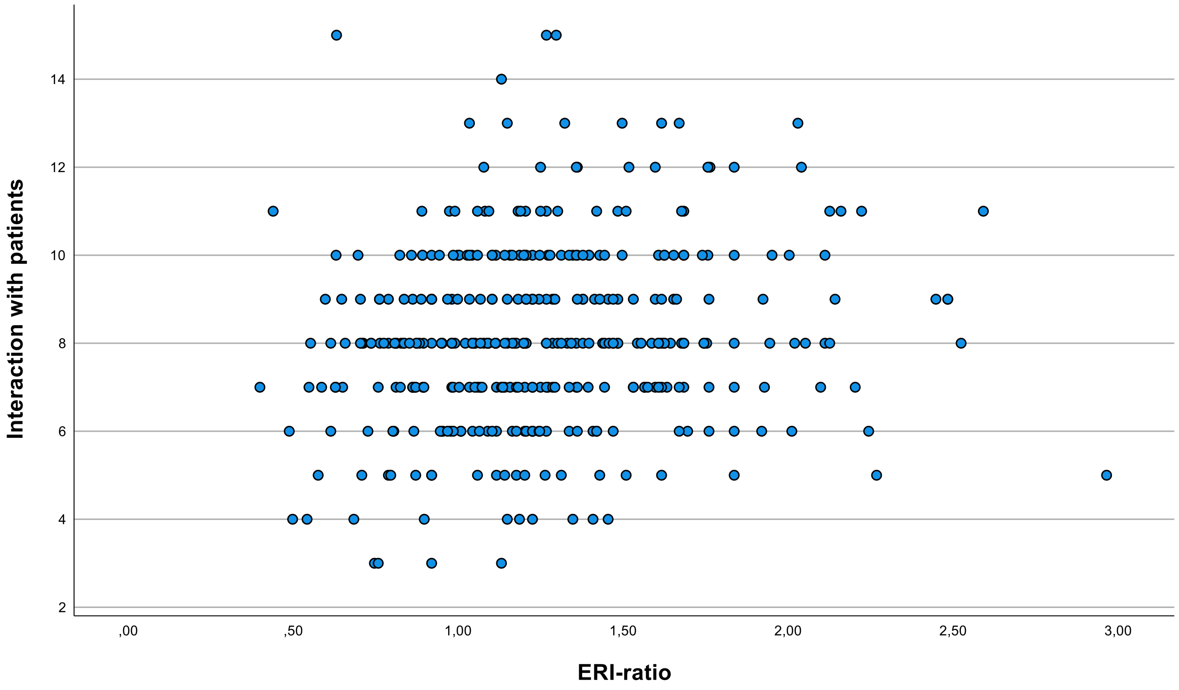

Figure S1.13. Scatterplot between effort-reward imbalance questionnaire (ERI)-ratio and poor interaction with patients. ERI=(Effort*11)/(Reward*6).

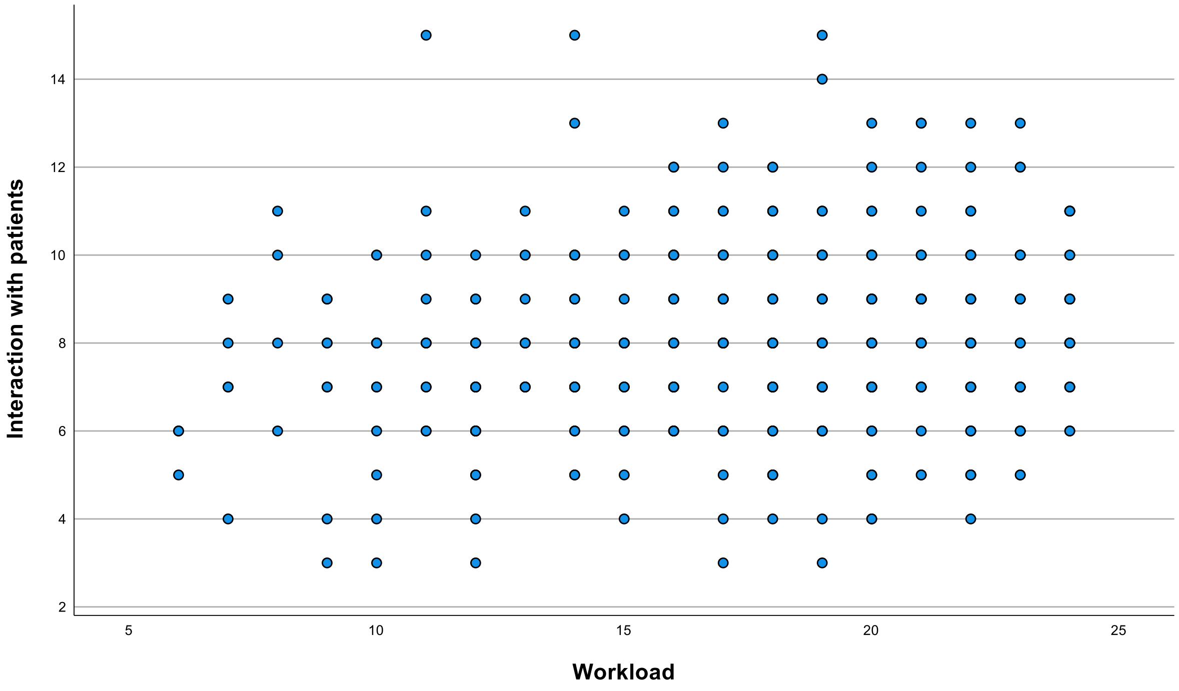

Figure S1.14. Scatterplot between workload and poor interaction with patients.

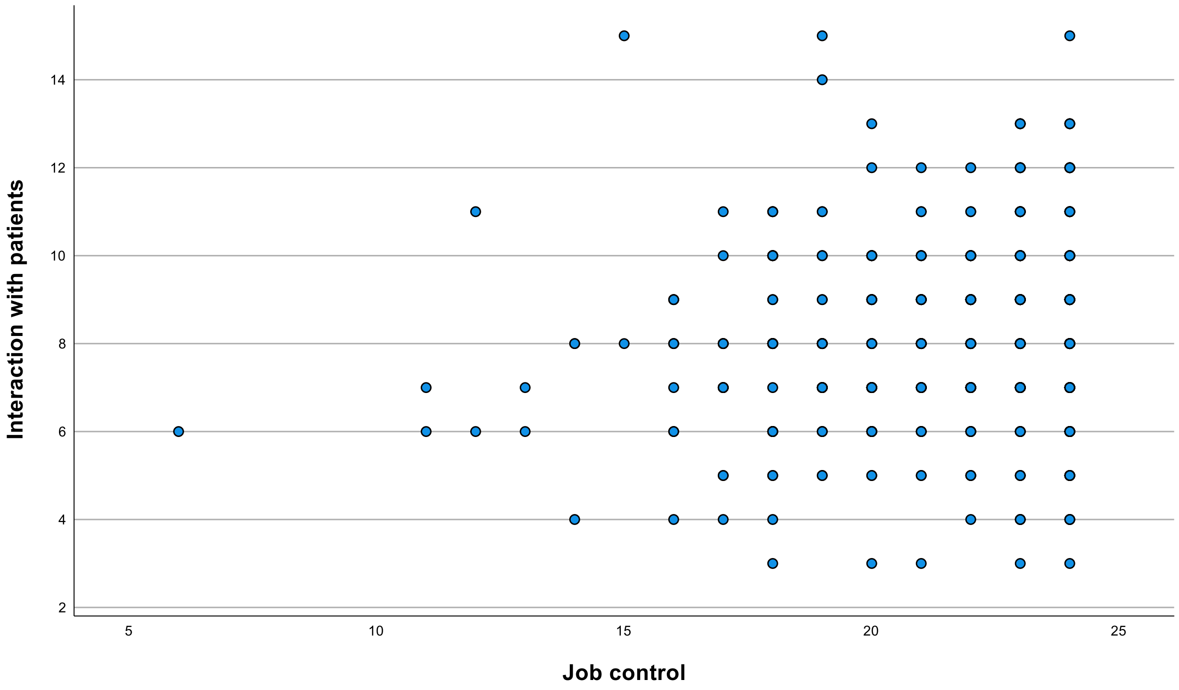

Figure S1.15. Scatterplot between job control and poor interaction with patients.

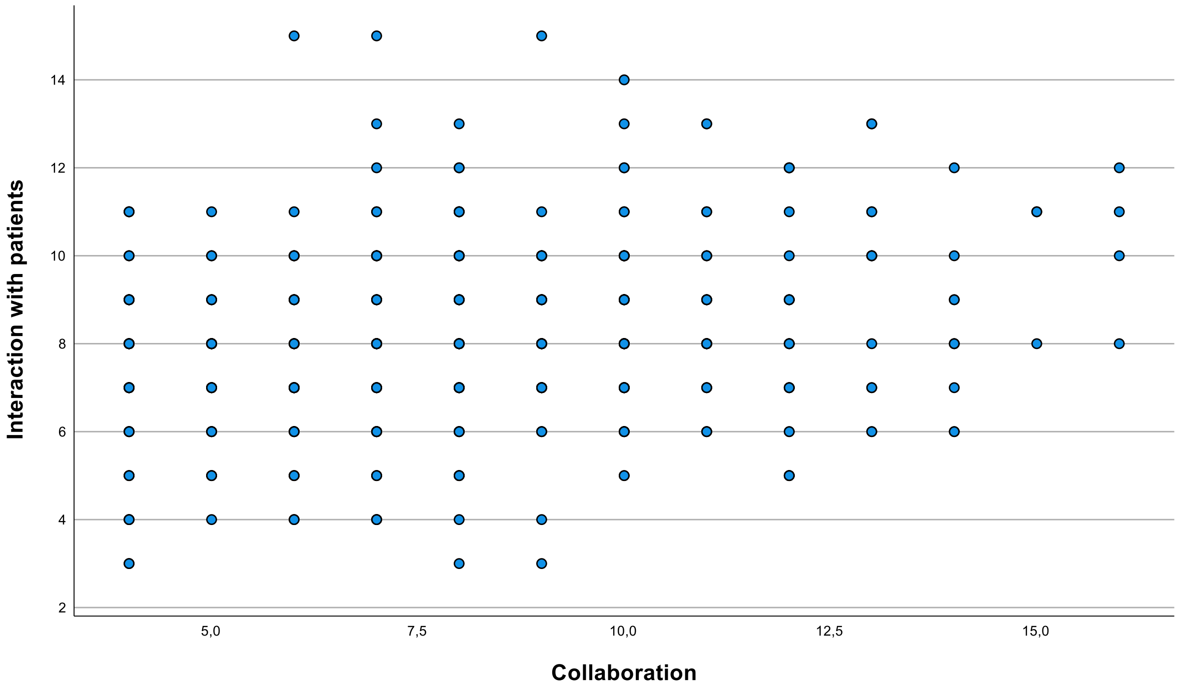

Figure S1.16. Scatterplot between collaboration and poor interaction with patients.

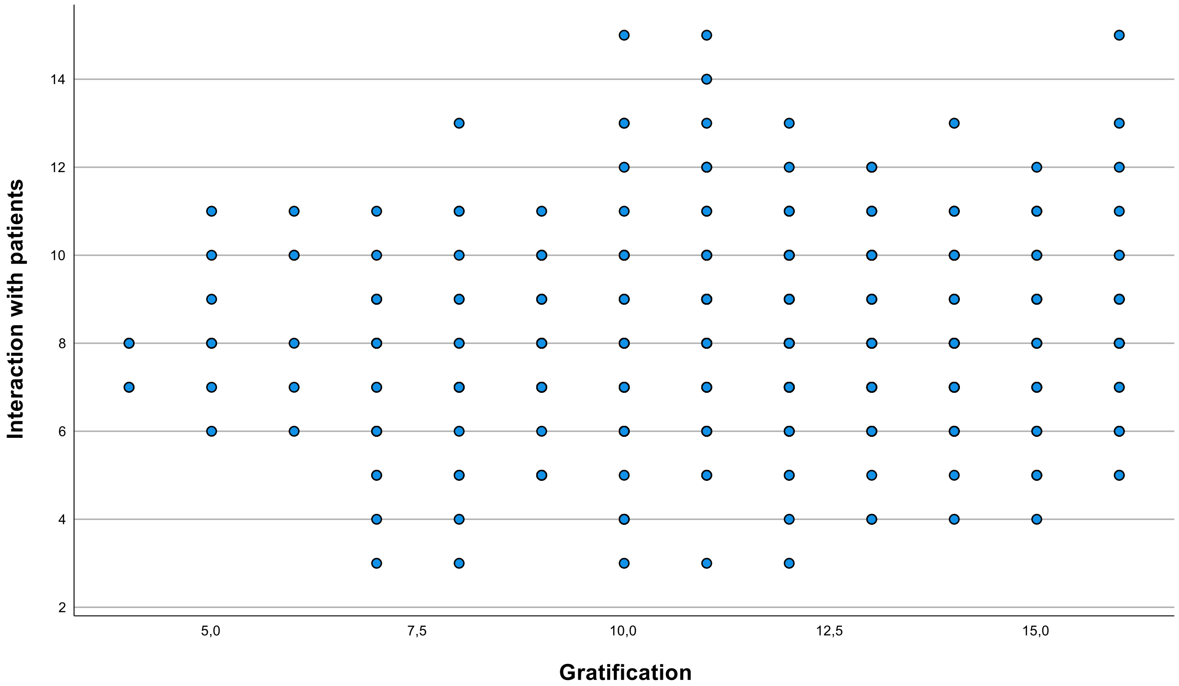

Figure S1.17. Scatterplot between gratification and poor interaction with patients.

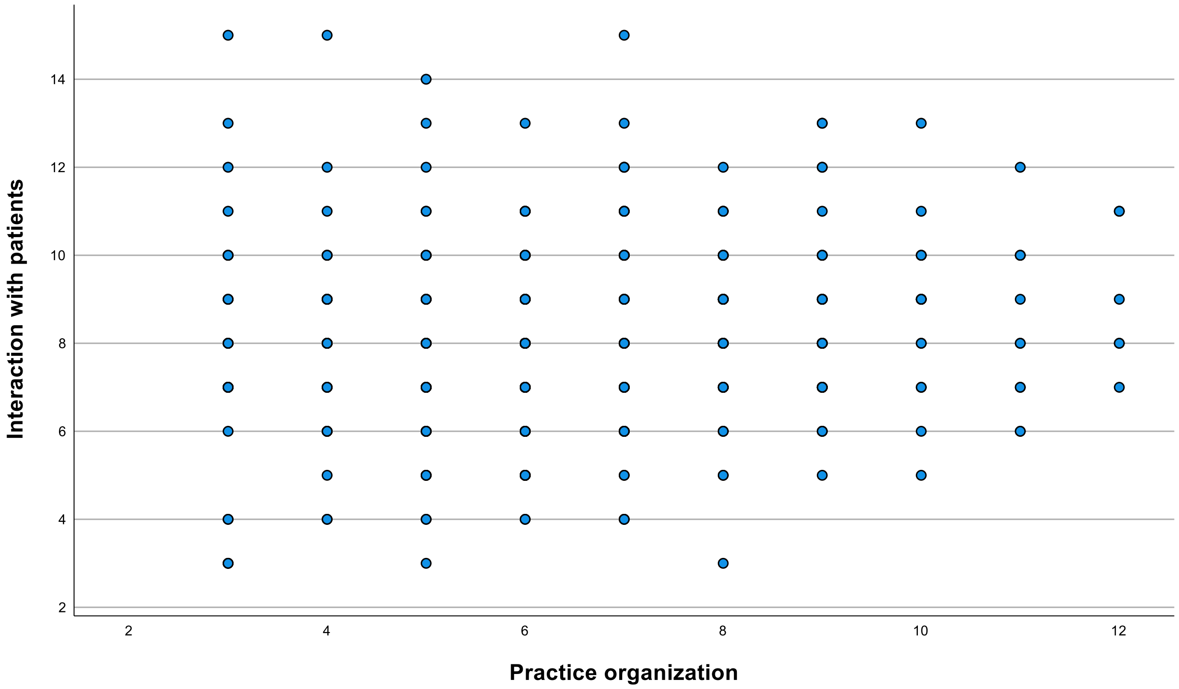

Figure S1.18. Scatterplot between practice organization and poor interaction with patients.

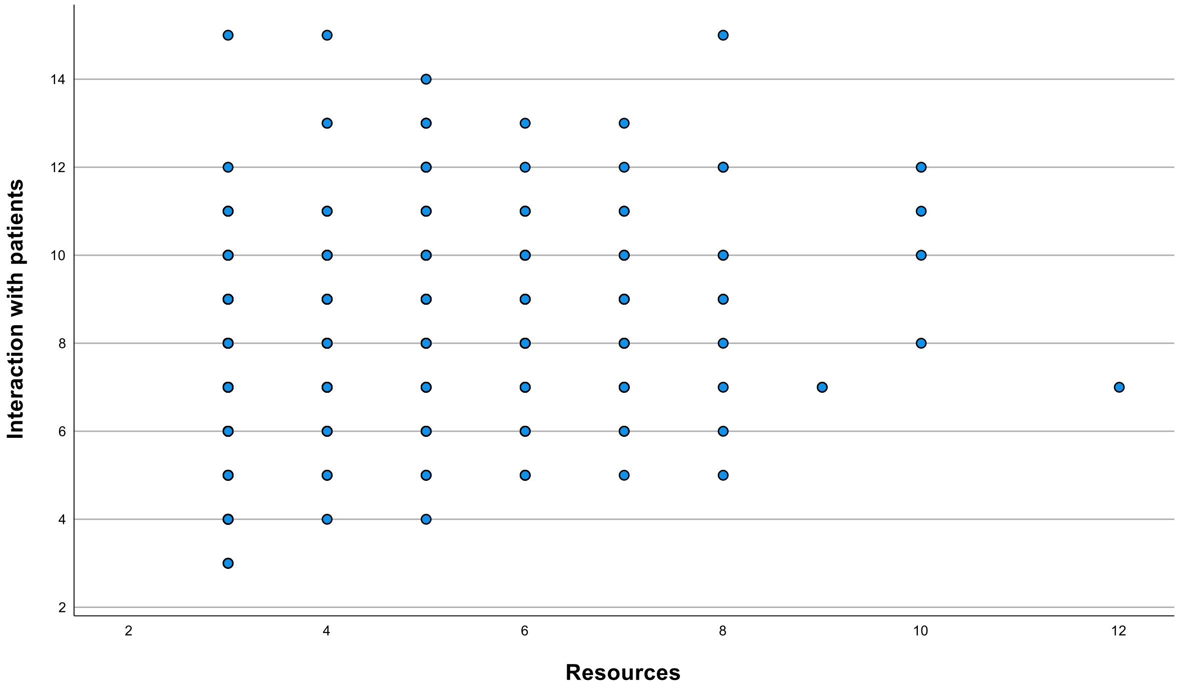

Figure S1.19. Scatterplot between resources and poor interaction with patients.

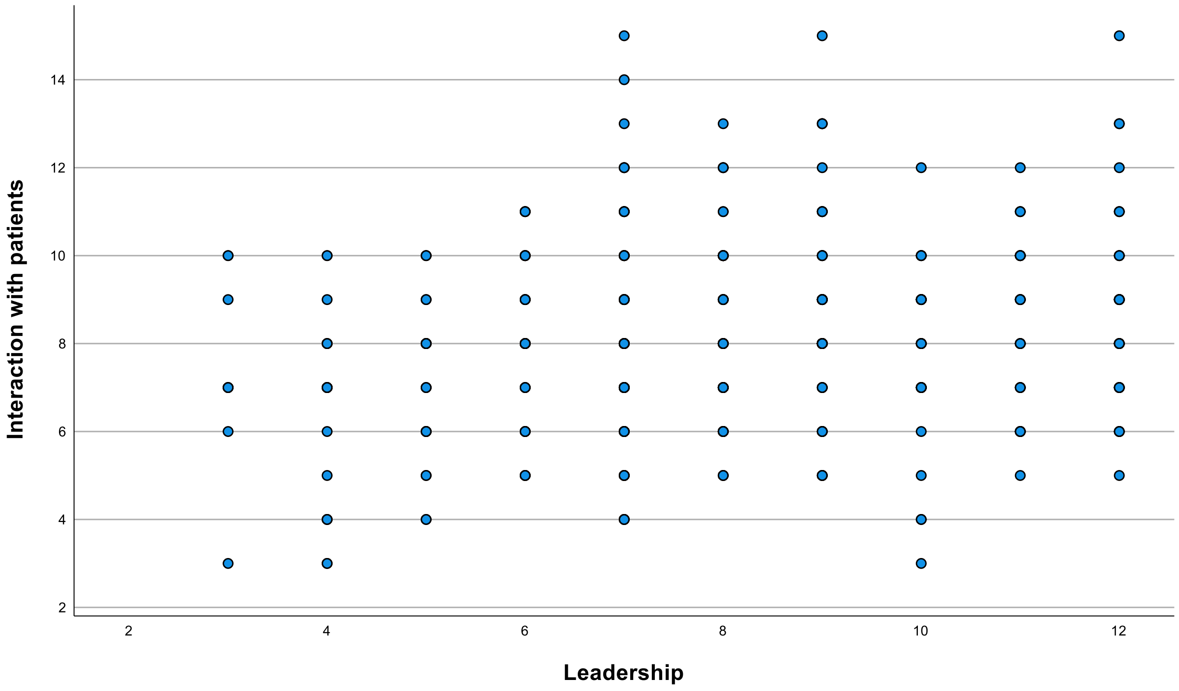

Figure S1.20. Scatterplot between leadership and poor interaction with patients.
