# Supplementary material for: Psychosocial working conditions as determinants of slips and lapses, and poor social interactions with patients among medical assistants in Germany: A cohort study
Source: PLoS One. 2024 Apr 16;19(4):e0296977. doi: 10.1371/journal.pone.0296977 (PMC11020507; doi:10.1371/journal.pone.0296977)
Supplement: S2 Table — (PDF) [file pone.0296977.s003.pdf]

Table S2. Detailed description of MA-specific working condition factors.

Factor	Psychosocial working condition factor	Examples	Number of items	Potential score range
1	Workload	Time pressure, staff shortage, high number of patients, working overtime	6	6-24
2	Job control	Documentation effort, unforeseeable events, interruptions and multitasking	6	6-24
3	Collaboration with supervisor/colleagues	Working climate, unfair treatment	4	4-16
4	Gratification	Career prospects, recognition, salary	4	4-16
5	Practice organization	Work structure, responsibilities, procedures	3	3-12
6	Resources	Interaction with patients, variety of work tasks, social character of the work	3	3-12
7	Leadership behavior	Recognition, work organization and conflict solving	3	3-12
