# Supplementary material for: Psychosocial working conditions as determinants of slips and lapses, and poor social interactions with patients among medical assistants in Germany: A cohort study
Source: PLoS One. 2024 Apr 16;19(4):e0296977. doi: 10.1371/journal.pone.0296977 (PMC11020507; doi:10.1371/journal.pone.0296977)
Supplement: S3 Table — (PDF) [file pone.0296977.s004.pdf]

Table S3.A: Model fit of the primary analysis between psychosocial working conditions and slips and lapses (see Table 2).

**Characteristic**		**Slips and lapses**									
		**Model I[a]**					**Model II[b]**				
		**B[c]**	**95%CI[d]**	**F-ratio**	**p-value**	**Adjusted R^2**	**B**	**95%CI**	**F-ratio**	**p-value**	**Adjusted R^2**
ERI model											
Effort	z-score[e]	-0.02	(-0.18, 0.14)	0.08	0.78	0.002	-0.00‘	(-0.16, 0.16)	0.15	0.932	-0.007
Reward	z-score	-0.18	(-0.34, -0.03)*	5.23	0.02	0.011	-0.18	(-0.34, -0.02)*	2.03	0.109	0.008
ERI-ratio	z-score	0.14	(-0.03, 0.30)	2.67	0.10	0.004	0.14	(-0.02, 0.31)	1.09	0.352	0.001
MA-specific instrument											
Workload (high)	z-score	-0.04	(-0.20, 0.11)	0.32	0.58	-0.002	-0.03	(-0.18, 0.12)	0.37	0.774	-0.005
Job control (low)	z-score	-0.07	(-0.22, 0.08)	0.79	0.38	-0.001	-0.06	(-0.21, 0.10)	0.43	0.736	-0.004
Collaboration (poor)	z-score	0.10	(-0.05, 0.26)	1.74	0.19	0.002	0.11	(-0.05, 0.27)	0.97	0.405	0.000
Gratification (low)	z-score	0.10	(-0.05, 0.26)	1.86	0.17	0.002	0.10	(-0.06, 0.25)	0.85	0.467	-0.001
Practice organization (poor)	z-score	0.11	(-0.05, 0.27)	1.96	0.16	0.002	0.12	(-0.04, 0.28)	1.13	0.337	0.001
Resources (lack of)	z-score	0.22	(0.06, 0.38)**	6.99	0.01	0.015	0.23	(0.07, 0.40)**	2.98	0.031	0.015
Leadership (poor)	z-score	0.13	(-0.03, 0.29)	2.42	0.12	0.004	0.13	(-0.03, 0.29)	1.20	0.309	0.002

Effort-reward imbalance questionnaire (ERI) or medical assistant (MA)-specific work stress questionnaire; for each exposure variable a separate regression model was computed; [a] unadjusted; [b] additionally adjusted for age and leadership position at baseline; [c] unstandardized coefficient; [d] confidence interval (CI); [e] a higher score reflects a higher agreement to the respective stressor; ‘exact B = 0.004, *p<0.05, **p<0.01, ***p<0.001

Table S3.B: Model fit of the primary analysis between psychosocial working conditions and poor interaction with patients (see Table 3).

Characteristic		Poor interaction with patients									
		Model I[a]					Model II[b]				
		B[c]	95%CI[d]	F-ratio	p-value	Adjusted R^2	B	95%CI	F-ratio	p-value	Adjusted R^2
ERI model											
Effort	z-score[e]	0.29	(0.09, 0.50)**	7.87	0.005	0.017	0.31	(0.10, 0.51)**	5.36	0.001	0.033
Reward	z-score	-0.33	(-0.53, -0.13)**	10.30	0.001	0.024	-0.32	(-0.52, -0.11)**	5.51	0.001	0.034
ERI-ratio	z-score	0.37	(0.15, 0.58)**	11.34	0.001	0.027	0.35	(0.14, 0.57)**	5.84	0.001	0.038
MA-specific instrument											
Workload (high)	z-score	0.32	(0.13, 0.52)**	10.41	0.001	0.024	0.33	(0.14, 0.53)**	5.95	0.001	0.037
Job control (low)	z-score	0.29	(0.09, 0.49)**	8.18	0.004	0.018	0.33	(0.13, 0.53)**	6.19	0.001	0.038
Collaboration (poor)	z-score	0.46	(0.27, 0.66)***	21.21	0.000	0.049	0.44	(0.24, 0.64)***	9.29	0.001	0.060
Gratification (low)	z-score	0.15	(-0.05, 0.34)	2.23	0.136	0.003	0.12	(-0.08, 0.32)	3.02	0.030	0.015
Practice organization (poor)	z-score	0.28	(0.08, 0.48)**	7.74	0.006	0.017	0.29	(0.08, 0.49)**	4.98	0.002	0.029
Resources (lack of)	z-score	[f]					[f]				
Leadership (poor)	z-score	0.41	(0.21, 0.61)***	15.76	0.000	0.036	0.41	(0.21, 0.62)***	7.95	0.001	0.050

Effort-reward imbalance questionnaire (ERI) or medical assistant (MA)-specific work stress questionnaire; for each exposure variable a separate regression model was computed;; [a] unadjusted; [b] additionally adjusted for age and leadership position at baseline; [c] unstandardized coefficient; [d] confidence interval (CI); [e] a higher score reflects a higher agreement to the respective stressor; [f] sub-scale "resources" removed from analysis from "poor interaction with patients" due to conceptual overlap; *p<0.05, **p<0.01, ***p<0.001
