# Supplementary material for: Psychosocial working conditions as determinants of slips and lapses, and poor social interactions with patients among medical assistants in Germany: A cohort study
Source: PLoS One. 2024 Apr 16;19(4):e0296977. doi: 10.1371/journal.pone.0296977 (PMC11020507; doi:10.1371/journal.pone.0296977)
Supplement: S4 Table — (PDF) [file pone.0296977.s005.pdf]

Table S4.A. Longitudinal associations of the ERI model and MA-specific stressors (z-scores) at baseline with slips and lapses at follow-up adjusted for potential intermediate factors among medical assistants (linear regression).

		Slips and lapses							
**Characteristic**		**Model II [a]**		**Model II + Vigor[b]**		**Model II + Dedication[c]**		**Model II + Work satisfaction[d]**	
		b	95%CI	b	95%CI	b	95%CI	b	95%CI
ERI model									
Effort	z-score	0.00	(-0.16, 0.16)	-0.07	(-0.23, 0.10)	-0.01	(-0.17, 0.14)	-0.08	(-0.25, 0.09)
Reward	z-score	-0.18	(-0.34, -0.02)*	-0.10	(-0.28, 0.08)	-0.10	(-0.28, 0.07)	-0.12	(-0.31, 0.07)
ERI	z-score	0.14	(-0.02, 0.31)	0.05	(-0.13, 0.23)	0.07	(-0.11, 0.25)	0.05	(-0.15, 0.25)
MA-specific instrument									
Workload	z-score	-0.03	(-0.18, 0.12)	-0.09	(-0.25, 0.06)	-0.06	(-0.21, 0.09)	-0.11	(-0.27, 0.06)
Job control	z-score	-0.06	(-0.21, 0.10)	-0.09	(-0.25, 0.07)	-0.05	(-0.20, 0.11)	-0.10	(-0.25, 0.06)
Collaboration	z-score	0.11	(-0.05, 0.27)	0.01	(-0.16, 0.19)	0.05	(-0.11, 0.22)	0.03	(-0.15, 0.22)
Gratification	z-score	0.10	(-0.06, 0.25)	0.04	(-0.13, 0.20)	0.03	(-0.14, 0.19)	0.04	(-0.13, 0.20)
Practice organization	z-score	0.12	(-0.04, 0.28)	0.05	(-0.12, 0.22)	0.07	(-0.10, 0.23)	0.06	(-0.11, 0.23)
Resources	z-score	0.23	(0.07, 0.40)**	0.16	(-0.03, 0.34)	0.19	(-0.01, 0.39)	0.19	(0.02, 0.36)*
Leadership	z-score	0.13	(-0.04, 0.28)	0.05	(-0.12, 0.23)	0.07	(-0.10, 0.24)	0.07	(-0.12, 0.24)

Effort-reward imbalance questionnaire (ERI) or medical assistant (MA)-specific work stress questionnaire; for each exposure variable a separate regression model was computed; [a] associations between adverse working condition exposures and the outcome poor patient interaction. model additionally adjusted for age and leadership position (Model II); [b] Model II additionally adjusted for vigor; [c] Model II additionally adjusted for dedication; [d] Model II additionally adjusted for work satisfaction. *p<0.05, **p<0.01;

Table S4.B. Longitudinal associations of the ERI model and MA-specific stressors (z-scores) at baseline with slips and lapses at follow-up adjusted for potential intermediate factors among medical assistants (linear regression).

		Slips and lapses							
**Characteristic**		**Model II [a]**		**Model II + Anxiety[b]**		**Model II + Depression[c]**		**Model II + Self-rated health[d]**	
		b	95%CI	b	95%CI	b	95%CI	b	95%CI
ERI model									
Effort	z-score	0.00	(-0.16, 0.16)	-0.02	(-0.18, 0.15)	-0.06	(-0.23, 0.11)	-0.04	(-0.20, 0.12)
Reward	z-score	-0.18	(-0.34, -0.02)*	-0.17	(-0.35, 0.00)	-0.09	(-0.28, 0.09)	-0.15	(-0.33, 0.02)
ERI	z-score	0.14	(-0.02, 0.31)	0.13	(-0.05, 0.31)	0.03	(-0.16, 0.22)	0.10	(-0.08, 0.28)
MA-specific instrument									
Workload	z-score	-0.03	(-0.18, 0.12)	-0.05	(-0.21, 0.12)	-0.08	(-0.24, 0.09)	-0.07	(-0.22, 0.09)
Job control	z-score	-0.06	(-0.21, 0.10)	-0.08	(-0.25, 0.08)	-0.11	(-0.27, 0.06)	-0.11	(-0.27, 0.05)
Collaboration	z-score	0.11	(-0.05, 0.27)	0.10	(-0.08, 0.27)	0.01	(-0.17, 0.20)	0.06	(-0.11, 0.23)
Gratification	z-score	0.10	(-0.06, 0.25)	0.08	(-0.08, 0.24)	0.04	(-0.13, 0.20)	0.08	(-0.08, 0.24)
Practice organization	z-score	0.12	(-0.04, 0.28)	0.10	(-0.06, 0.27)	0.06	(-0.11, 0.23)	0.07	(-0.10, 0.24)
Resources	z-score	0.23	(0.07, 0.40)**	0.22	(0.05, 0.38)*	0.18	(0.01, 0.36)*	0.19	(0.03, 0.36)*
Leadership	z-score	0.13	(-0.03, 0.29)	0.11	(-0.06, 0.29)	0.06	(-0.12, 0.24)	0.08	(-0.10, 0.25)

Effort-reward imbalance questionnaire (ERI) or medical assistant (MA)-specific work stress questionnaire; for each exposure variable a separate regression model was computed; [a] associations between adverse working condition exposures and the outcome poor patient interaction. [b]Model II additionally adjusted for anxiety (GAD2); [c] Model II additionally adjusted for depression (PHQ2); [d] Model II additionally adjusted for self-rated health. *p<0.05, **p<0.01;

Table S4.C. Longitudinal associations of the ERI model and MA-specific stressors (z-scores) at baseline with poor patient interaction at follow-up adjusted for potential intermediate factors among medical assistants (linear regression).

		Poorer patient interaction							
**Characteristic**		**Model II** [a]		**Model II + Vigor**[b]		**Model II + Dedication**[c]		**Model II + Work satisfaction**[d]	
		b	95%CI	b	95%CI	b	95%CI	b	95%CI
ERI model									
Effort	z-score	0.31	(0.10, 0.51)**	0.17	(-0.03, 0.37)	0.26	(0.07, 0.46)**	0.22	(0.00, 0.43)
Reward	z-score	-0.32	(-0.52, -0.11)**	-0.08	(-0.30, 0.14)	-0.12	(-0.34, 0.10)	-0.21	(-0.46, 0.03)
ERI	z-score	0.35	(0.14, 0.57)**	0.10	(-0.13, 0.33)	0.19	(-0.03, 0.41)	0.23	(-0.03, 0.48)
MA-specific instrument									
Workload	z-score	0.33	(0.14, 0.53)**	0.18	(-0.01, 0.38)	0.24	(0.05, 0.44)*	0.25	(0.04, 0.47)*
Job control	z-score	0.33	(0.13, 0.53)**	0.26	(0.07, 0.45)**	0.32	(0.13, 0.52)***	0.28	(0.08, 0.49)**
Collaboration	z-score	0.44	(0.24, 0.64)***	0.24	(0.02, 0.45)*	0.31	(0.11, 0.52)**	0.39	(0.16, 0.62)***
Gratification	z-score	0.12	(-0.08, 0.32)	-0,04	(-0.24, 0.16)	-0,05	(-0.25, 0.16)	0,00	(-0.21, 0.22)
Practice organization	z-score	0.29	(0.08, 0.49)**	0.11	(-0.10, 0.32)	0.13	(-0.07, 0.34)	0.19	(-0.03, 0.41)
Leadership	z-score	0.41	(0.21, 0.62)***	0.23	(0.02, 0.45)*	0.27	(0.06, 0.48)*	0.34	(0.11, 0.57)**

Effort-reward imbalance questionnaire (ERI) or medical assistant (MA)-specific work stress questionnaire; for each exposure variable a separate regression model was computed; sub-scale “resources” removed from analysis from “poor interaction with patients” due to conceptual overlap; [a] associations between adverse working condition exposures and the outcome poor patient interaction. model additionally adjusted for age and leadership position (Model II); [b] Model II additionally adjusted for vigor; [c] Model II additionally adjusted for dedication; [d] Model II additionally adjusted for work satisfaction. *p<0.05, **p<0.01; ***p<0.001

Table S4.D. Longitudinal associations of the ERI model and MA-specific stressors (z-scores) at baseline with poor patient interaction at follow-up adjusted for potential intermediate factors among medical assistants (linear regression).

		Poorer patient interaction							
**Characteristic**		**Model II [a]**		**Model II + Anxiety[b]**		**Model II + Depression[c]**		**Model II + Self-rated health[d]**	
		b	95%CI	b	95%CI	b	95%CI	b	95%CI
ERI model									
Effort	z-score	0.31	(0.10, 0.51)**	0.21	(0.01, 0.42)*	0.20	(-0.02, 0.41)	0.23	(0.03, 0.44)*
Reward	z-score	-0.32	(-0.52, -0.11)**	-0.20	(-0.42, 0.02)	-0.18	(-0.41, 0.05)	-0.25	(-0.46, -0.03)*
ERI	z-score	0.35	(0.14, 0.57)**	0.22	(-0.01, 0.44)	0.21	(-0.03, 0.45)	0.31	(0.08, 0.54)**
MA-specific instrument									
Workload	z-score	0.33	(0.14, 0.53)**	0.23	(0.03, 0.43)*	0.21	(0.00, 0.41)*	0.24	(0.04, 0.44)*
Job control	z-score	0.33	(0.13, 0.53)**	0.25	(0.04, 0.45)*	0.25	(0.04, 0.45)*	0.27	(0.07, 0.47)**
Collaboration	z-score	0.44	(0.24, 0.64)***	0.33	(0.11, 0.55)**	0.32	(0.09, 0.55)**	0.34	(0.13, 0.56)**
Gratification	z-score	0.12	(-0.08, 0.32)	0,04	(-0.16, 0.25)	0,02	(-0.19, 0.23)	0,07	(-0.13, 0.27)
Practice organization	z-score	0.29	(0.08, 0.49)**	0.19	(-0.02, 0.40)	0.19	(-0.02, 0.41)	0.20	(-0.01, 0.42)
Leadership	z-score	0.41	(0.21, 0.62)***	0.31	(0.10, 0.52)**	0.28	(0.06, 0.50)*	0.32	(0.11, 0.53)**

Effort-reward imbalance questionnaire (ERI) or medical assistant (MA)-specific work stress questionnaire; for each exposure variable a separate regression model was computed; sub-scale "resources" removed from analysis from "poor interaction with patients" due to conceptual overlap; [a] associations between adverse working condition exposures and the outcome poor patient interaction. [b]Model II additionally adjusted for anxiety (GAD2); [c] Model II additionally adjusted for depression (PHQ2); [d] Model II additionally adjusted for self-rated health. *p<0.05, **p<0.01; ***p<0.001
