# Supplementary material for: Psychosocial working conditions as determinants of slips and lapses, and poor social interactions with patients among medical assistants in Germany: A cohort study
Source: PLoS One. 2024 Apr 16;19(4):e0296977. doi: 10.1371/journal.pone.0296977 (PMC11020507; doi:10.1371/journal.pone.0296977)
Supplement: Attachment — Submitted filename: Responses to reviewers.pdf [file pone.0296977.s006.pdf]

Reviewer Comments:

Reviewer #1: The authors have investigated a subject of great importance to the quality of interaction between clinicians and patients. The study has been well presented. You may want to read lines 509 and 510 to clarify the intended point. Congratulations

Response: We thank you Reviewer 1 for the positive review. As recommended, we corrected the sentence in line 509 and 510 to clarify the point we sought to make:

Line 541-542: “Moreover, we suggest that a promising starting point is to improve communication across hierarchical structures.”

Reviewer #2: None

Response:
Not applicable.

Reviewer #3: This is a very interesting cross-sectional research on potential associations for working conditions and slips/lapses for medical assistants. This was an extremely difficult study to execute, even moreso with the pandemic. The paper is very well-written and nicely mentions the need for more objective measures of slips and lapses. My main comments are about the analyses and the the possibility there could be restriction of range in the data, particularily for slips and lapses.

Response:
We thank Reviewer 3 very much for the thorough review of our manuscript and for the very constructive comments and suggestions that helped us to substantially improve the manuscript.

Major Comments
1) Regression models
a) Overall model fit: Suggest using the F-ratio, p-value, and adjusted R2

Response:

We calculated the suggested model fit indicators. As associations between the psychosocial working conditions and the outcome slips and lapses were mostly non-significant, the corresponding F-ratios were not significant and adjusted R^2 were very low. Associations between psychosocial working conditions and poor interaction with patients showed higher and significant F-ratios, lower p-values and higher adjusted R^2.

In public health research, it is common practice to focus mainly on the association measures with the corresponding p-values or 95% confidence intervals rather than on the overall model fit, as we do not seek to identify the best model based on sets of variables, but aim to examine single exposures of interest. This is in line with our approach to calculate separate models for all combinations of psychosocial working conditions and outcome variables. We therefore now present the model fit indicators (F-ratio, p-value and adjusted R^2) for the primary analysis between exposures and outcomes in the appendix 'S3 Table' and refer to the appendix in the footnote of table 2 and table 3.

b) Correlation matrix: I recommend showing all the correlations among variables before the regression models. My concern is there could be high collinearity in the regression models.

Response:

We perfectly understand the reviewer's concerns. Please find the suggested correlation matrix below. Multicollinearity among the exposure variables (or independent variables) may induce inflated standard errors and thereby biased estimations – given that the relevant exposure variables are included in the same regression model.

Notably though, in our study, we ran a separate regression model for each combination of exposure and outcome variable. Therefore, multicollinearity cannot have affected our findings. The fully-adjusted models only included age and leadership position as additional variables. We now explain better in the method section that regression models were computed separately for each exposure variable.

Moreover, we now specifically state in the footnotes of each table presenting regression model results that we computed for each exposure variable a separate regression model.

Line 231-234: "Associations were quantified using unstandardized regression coefficients (B) and corresponding 95% confidence intervals (95% CIs), which were estimated based on multivariable linear regressions. Regression models were computed separately for each combination of exposure variable and outcome variable."

Line 316-317 and 336-337: Footnote in Table 2 and Table 3: “for each exposure variable a separate regression model was computed […].”

1 Table. Correlation matrix (Pearson correlation) of dependent, independent and potential mediating variables.

	IP	SL	EF	RW	ERI	WL	JC	CB	GF	PO	RS	LS	VI	DD	WS	AX	DP	SRH
IP	1																	
SL	,305**	1																
EF	,121*	-,014	1															
RW	-,155**	-,105*	-,338**	1														
ERIi	,157**	,076	,722**	-,844**	1													
WL	,152**	-,028	,839**	-,398**	,660**	1												
JC	,115*	-,045	,770**	-,202**	,506**	,623**	1											
CB	,224**	,064	,450**	-,658**	,684**	,501**	,308**	1										
GF	,079	,056	,660**	,245**	-,838**	,295**	,099*	,385**	1									
PO	,128**	,072	,302**	-,399**	,428**	,413**	,180**	,488**	,195**	1								
RS	,215**	,133**	,225**	,042	-,273**	,099*	-0,013	,255**	,202**	,197**	1							
LS	,196**	,077	,383**	-,671**	,635**	,465**	,269**	,582**	,450**	,524**	,183**	1						
VI	-,298**	-,132**	-,213**	,472**	-,439**	-,284**	-,088	-,469**	-,313**	-,355**	-,477**	-,385**	1					
DD	-,264**	-,126*	-,089	,430**	-,327**	-,209**	-,028	-,318**	-,315**	-,293**	-,571**	-,333**	,784**	1				
WS	-,172**	-,107*	-,308**	,562**	-,559**	-,384**	-,134**	-,512**	-,409**	-,397**	-,305**	-,446**	,559**	,492**	1			
AX	,192**	,044	,207**	-,379**	,350**	,265**	,166**	,426**	,221**	,241**	,160**	,311**	-,421**	-,258**	-,417**	1		
DP	,222**	,100*	,250**	-,450**	,451**	,297**	,151**	,477**	,295**	,277**	,263**	,384**	-,548**	-,414**	-,564**	,668**	1	
SRH	,179**	,051	,229**	-,329**	,335**	,264**	,133**	,371**	,214**	,313**	0,082	,322**	-,441**	-,236**	-,375**	,428**	,399**	1

** $p < 0.01$ (two-tailed), * $p < 0.05$ (two-tailed)

IP, interaction with patients; SL, slips and lapses; EF, effort; RW, reward; ERI, ERI ratio; WL, Workload; JC, Job control; CB, Collaboration; GF, Gratification; PO, Practice organization, RS, Resources; LS, Leadership; VI, vigor; DD, dedication; WS, work satisfaction; AX, anxiety; DP, depression; SRH, self-rated health;

c) Mediation analysis: "Mediation analysis was performed for significant associations between exposure and outcomes." This may be problematic because mediation can still occur with non-significant associations, see https://www.ncbi.nlm.nih.gov/pmc/articles/PMC2819361
- I'm definitely not an expert in mediation, but my limited understanding is the current best practice is to use bootstrapping: https://www.processmacro.org/index.html
Preacher and Hayes have many publications about this- https://www.processmacro.org/papers.html

Response:
We highly value your input and literature suggestions. For the first time, we aimed to explore potential intermediate factors in the association between psychosocial working conditions and 'slips and lapses' and 'poor interaction with patients'. An exploratory approach was applied to identify potential intermediate factors. The chosen statistical approach, that is, the 'difference method', is an approach commonly used in epidemiological research [1]. However, we refrained from reporting detailed estimates for the indirect effects and instead showed trends. We aimed to identify patterns regarding potential intermediate factors in order to establish direction of further research in light of the sparse evidence [1].
We sought to emphasize the exploratory approach towards the readership by consistently referring to "potential intermediate factors" rather than mediators in the text. To clarify that an explorative approach using the difference method was applied, we now refer to these two aspects in the statistical analysis section of the materials and methods section (see below).

Moreover, we thank the reviewer for pointing out the importance of reporting the regression estimates of the mediation analysis of the non-significant associations between exposure and outcomes due to the possibility of potential mediation [2]. We now report regression estimates of potential intermediate factors for significant and non-significant associations between exposures and outcomes in the Tables A, B, C, and D in the appendix S4 Table and refer to those tables in the result section.
Due to the exploratory approach and the common application in epidemiological research, we would therefore like to retain our original analytical approach using the difference method as shown in the manuscript.

However, based on your valuable input we now discuss in the 'recommendations for future research' section, that future research should further investigate and validate the identified potential intermediate factors by applying statistical approaches such as the process macro by Hayes [3].

Moreover, based on the literature you recommended [2], we now state in the limitation section that confounding and mediation differentiate based on conceptual consideration and therefore, the identified potential intermediate factors may as well be confounders.

Line 242-248: "To explore potential intermediate factors, the corresponding variables were added separately to the regression models as continuous variables (i.e., vigor, dedication, depression, and anxiety) or ordinal variables (i.e., work satisfaction and self-rated health). Indication of mediation was considered to be present if the association between a given exposure and a given outcome was attenuated towards the null value (B=0) after adjustment for the respective potential intermediate factor. This approach is referred to as the difference method (39)."

Line 343-347: "Mediation analysis was performed for both significant and non-significant associations between exposures and outcomes (see S4 Table). Focusing on significant associations from the primary analysis, we observed an attenuation towards the null value (B=0) for the association of reward with slips and lapses after adjustment for work engagement and depression. […]"

Line 519-521: "Finally, the differentiation between mediators and cofounders is based on conceptual considerations [53]. Therefore, the attenuation of observed associations could also be due to confounding rather than mediation."

Line 531-533: "Moreover, in an exploratory approach, we applied the difference method to explore potential intermediate factors. Future research could examine potential mediation in greater detail [56].

References:

1) VanderWeele TJ. Mediation analysis: a practitioner's guide. Annual review of public health. 2016;37:17-32.
2) MacKinnon DP, Krull JL, Lockwood CM. Equivalence of the mediation, confounding and suppression effect. Prevention science. 2000:173-81.
3) Hayes AF. Introduction to mediation, moderation, and conditional process analysis: A regression-based approach. Guilford publications; 2017.

d) Unit of analysis: It appears that each medical assistant was treated as an independent unit of analysis? Were there medical assistants from the same clinics, same regions? If

so, cluster adjusted standard errors/variance should be used (or multilevel modeling). This may or may not make much of a difference in the results. I'm not sure about using cluster adjustment with mediation.

Response:
Thank you for highlighting this interesting point. We agree that multi-level analyses would be a suitable approach, if we had included several medical assistants from the same health care institutions. Unfortunately, we do not have data that could reveal such clustering. The likelihood of such clustering to a significant extent is low however, as we recruited a random sample of medical assistants from all over Germany and did not approach participants through their employers.

We now added the following to the limitation section:

Line 490-494: “We did not collect data on the MAs’ employer and thus do not know whether we included MAs who work for the same employer. In case of such clustering of observations, different statistical approaches had been preferable (e.g., multilevel modeling). However, such clustering to a large extent seems unlikely as we recruited our sample of 408 MAs from all over Germany and not primarily through their employer.”

1) **Restriction of range? Could limited ranges of responses be impacting the regression results? For example, for work satistifaction: only 2.45% of MA were very unsatisified and only 11.5% were very unsatisified. Slips and lapses appeared to be at the lower end of the range [mean = 5.45 with a min= 3], so they are at least somewhat rare? If this is the case, acknowledging it as a limitation should be sufficient.**

Response:
We agree with you and as recommended we now mention the restriction of range in the limitation section.

Line 495-496: “The distribution of some variables may have displayed a restricted range (e.g., slips and lapses, work satisfaction), which may have impacted the regression estimates.”

Minor Comments
1) Graphs: It'd be really helpful to see scatter plots among the different variables. Perhaps this could be reported in the appendix?

Response:

As proposed, we now provide the scatterplots between the exposure variables and outcome variables in appendix S1 Figure and refer to these in the result section.

Line 256-257: “The scatterplots for exposure variables and outcome variables can be found in S1 Figure.”

2) Data availability: I understand privacy concerns, but would partial data-sharing be possible? Such as the individual survey data without demographic and/or other information about the Medical Assistants that might be used for re-identification?

Response:

We fully support data sharing to increase transparency in science. However, the study information and the informed consent form signed by participants’ state that data will not be shared. We conferred with the data security officer at our institution and were told that we may still share data – in case the data is anonymized and shared with research institutions in the European Union (due to uniform and strict data protection regulations within the European Union). Therefore, general data sharing is unfortunately not feasible and only available on reasonable request.
